# Understanding the Cholera Epidemic, Haiti

**DOI:** 10.3201/eid1707.110059

**Published:** 2011-07

**Authors:** Renaud Piarroux, Robert Barrais, Benoît Faucher, Rachel Haus, Martine Piarroux, Jean Gaudart, Roc Magloire, Didier Raoult

**Affiliations:** Author affiliations: Université de la Méditerranée, Marseilles, France (R. Piarroux, B. Faucher, J. Gaudart, D. Raoult);; Ministère de la Santé Publique et de la Population, Port-au-Prince, Haiti (R. Barrais, R. Magloire);; Service de Santé des Armées, Paris, France (R. Haus);; Martine Piarroux Université de Franche-Comté, Besançon, France (M. Piarroux)

## Abstract

After onset of a cholera epidemic in Haiti in mid-October 2010, a team of researchers from France and Haiti implemented field investigations and built a database of daily cases to facilitate identification of communes most affected. Several models were used to identify spatiotemporal clusters, assess relative risk associated with the epidemic’s spread, and investigate causes of its rapid expansion in Artibonite Department. Spatiotemporal analyses highlighted 5 significant clusters (p<0.001): 1 near Mirebalais (October 16–19) next to a United Nations camp with deficient sanitation, 1 along the Artibonite River (October 20–28), and 3 caused by the centrifugal epidemic spread during November. The regression model indicated that cholera more severely affected communes in the coastal plain (risk ratio 4.91) along the Artibonite River downstream of Mirebalais (risk ratio 4.60). Our findings strongly suggest that contamination of the Artibonite and 1 of its tributaries downstream from a military camp triggered the epidemic.

On October 21, 2010, the Haitian Ministry of Public Health and Population (MSPP) reported a cholera epidemic caused by *Vibrio cholerae* O1, serotype Ogawa, biotype El Tor (*1*). This epidemic was surprising as no cholera outbreak had been reported in Haiti for more than a century (*1**,**2*). Numerous media rapidly related the epidemic to the deadly earthquake that Haiti had experienced 9 months earlier. However, simultaneously, a rumor held recently incoming Nepalese soldiers responsible for importing cholera, along with accusations of illegal dumping of waste tank contents (*3*). A cholera outbreak was indeed reported in Nepal’s capital city of Kathmandu on September 23, 2010, shortly before troops left for Haiti (*4**,**5*). Two hypotheses then emerged to explain cholera in Haiti.

Some researchers posited the transmission of an environmental strain to humans (*6*). Reasoning by analogy with cholera epidemiology in South Asia, they hypothesized that weather conditions, i.e., the La Niña phenomenon, might have promoted the growth of *V. cholerae* in its environmental reservoir (*6*). The second hypothesis suggested importation of the disease from a cholera-endemic country. The sequencing of 2 isolates of *V. cholerae* supported this second hypothesis by establishing an exogenous origin, probably from southern Asia or eastern Africa (*7*). Responding to a request from Haitian authorities to the French Embassy for the support of epidemiologists, we conducted a joint French–Haitian investigation during November 7–November 27, 2010, to clarify the source of the epidemic and its unusual dynamic.

## Morbidity and Mortality Survey

As soon as the epidemic was recognized, a nationwide monitoring program was implemented to register all ambulatory patients, hospital admissions, and deaths (*1*). Each day, all government and nongovernmental health facilities in Haiti reported cases to the Direction of Health in each department, which colligated data before sending them to MSPP. For this study, the departments were asked to provide more precise data corresponding to the 140 Haitian communes from October 16 through November 30. Probable cholera cases were defined as profuse, acute watery diarrhea in persons. In each department, bacteriologic confirmation was obtained only for the first cases. Children <5 years of age were included because age was not always reported. Community deaths were additionally reported by local authorities. Comparison with epidemiologic surveys performed by other actors (Doctors without Borders, medical brigades from Cuba) enabled confirmation of the consistency of the database. Cholera incidence was calculated by using population numbers from Haitian authorities and mapped together with environmental settings by using ArcGIS (ESRI, Redlands, CA, USA). Maps of gridded population density (*8*), communes, rivers, roads, altitude, internally displaced persons (IDP) camps, and health facilities were obtained from Haitian authorities and the United Nations Stabilization Mission in Haiti (MINUSTAH) website (http://minustah.org).

## Field Surveys

The first team of epidemiologists from Haiti went to Mirebalais during October 19–24. Then, from November 7–27, epidemiology teams from France and Haiti visited the most affected areas, namely Mirebalais, St-Marc, Gonaïves, Cap Haïtien, St-Michel-de-l’Attalaye, Petite-Rivière-de-l’Artibonite, Ennery, Plaisance, and Port-au-Prince. These visits included interviews with health actors and civilian authorities and investigation of environmental risks among inhabitants and patients from cholera treatment centers.

## Statistics

To investigate for space–time case clustering, we analyzed the daily case numbers in each Haitian commune from October 16 through November 30 using SaTScan software (Kulldorf, Cambridge, UK). To detect clusters, this software systematically moves a circular scanning window of increasing diameter over the studied region and compares observed case numbers inside the window to the numbers that would be expected under the null hypothesis (random distribution of cases). The maximum allowed cluster size corresponded to 50% of the Haitian population. The statistical significance for each cluster was obtained through Monte Carlo hypothesis testing, i.e., results of the likelihood function were compared with 999 random replications of the dataset generated under the null hypothesis (*9**,**10*).

On the basis of these results, we further analyzed risk factors for spread in Ouest, Centre, and Artibonite Departments during October 20–28 using a regression model with adjustment on spatial variability. The initial focus, Mirebalais, was precluded to better estimate the relationship between the epidemic spread and the distance to the epidemic source. Because data on cholera cases were non-normally distributed and thus violated basic assumptions for linear regression, we used a generalized additive model (GAM) (*11**–**13*). Furthermore, because of the over-dispersion of the data (variance was greater than mean), we used a quasi-Poisson model (variance = *c* × mean, where *c* is an estimated constant) (*14*). The use of a Poisson model would not have been relevant because the main assumption for Poisson models is that variance equals mean. The GAM was allowed to model the count of cases in each commune, analyzing 1 continuous variable (distance to Mirebalais) and 3 binary variables (location downstream of Meille River, presence of camps of IDP, and commune partially or totally located in coastal plain). The models were adjusted on the population and the spatial distribution of communes. Conditions of use were checked by using classical graphic means. The goodness-of-fit was also assessed by the percentage of explained deviance.

In the communes bordering the Artibonite River, namely Mirebalais, St-Marc, Dessalines, Petite-Rivière-de-l’Artibonite, Grande Saline, Verrettes, Desdunes, and L’Estère, during October 16–31, we searched for synchronizations between communal epidemiologic curves by calculating and testing Spearman correlation coefficients. Statistical analyses were performed by using R version 2.10.1 software (www.r-project.org/foundation), particularly with the *mgcv* package (GAM modeling) (*11*). We compared p values to the probability threshold α = 0.05.

## Initiation

**Figure 1:**
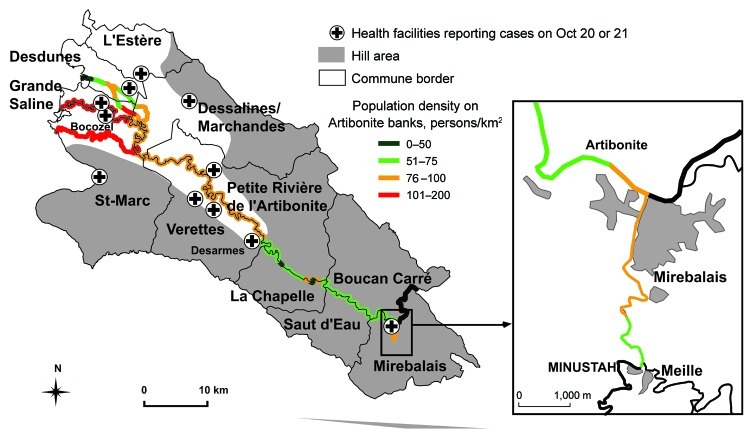
Location of health centers reporting cholera cases in communes along the Artibonite River on October 20, 2010, Haiti. MINUSTAH, United Nations Stabilization Mission in Haiti.

On October 18, the Cuban medical brigades reported an increase of acute watery diarrhea (61 cases treated in Mirebalais during the preceding week) to MSPP. On October 18, the situation worsened, with 28 new admissions and 2 deaths. MSPP immediately sent a Haitian investigation team, which found that the epidemic began October 14. The first hospitalized patients were members of a family living in Meille (also spelled Méyè), a small village 2 km south of Mirebalais (Figure 1). On October 19, the investigators identified 10 other cases in the 16 houses near the index family’s house. Five of the 6 samples collected in Meille from these outpatients, who became sick during October 14–19, yielded *V. cholerae* O1, serotype Ogawa, biotype El Tor. Environmental and water source samples proved negative.

Meille village hosted a MINUSTAH camp, which was set up just above a stream flowing into the Artibonite River. Newly incoming Nepalese soldiers arrived there on October 9, 12, and 16. The Haitian epidemiologists observed sanitary deficiencies, including a pipe discharging sewage from the camp into the river. Villagers used water from this stream for cooking and drinking.

On October 21, the epidemic was also investigated in several wards of Mirebalais. Inhabitants of Mirebalais drew water from the rivers because the water supply network was being repaired. Notably, prisoners drank water from the same river, downstream from Meille. No other cause was found for the 34 cases and 4 deaths reported in the prison.

**Figure 2:**
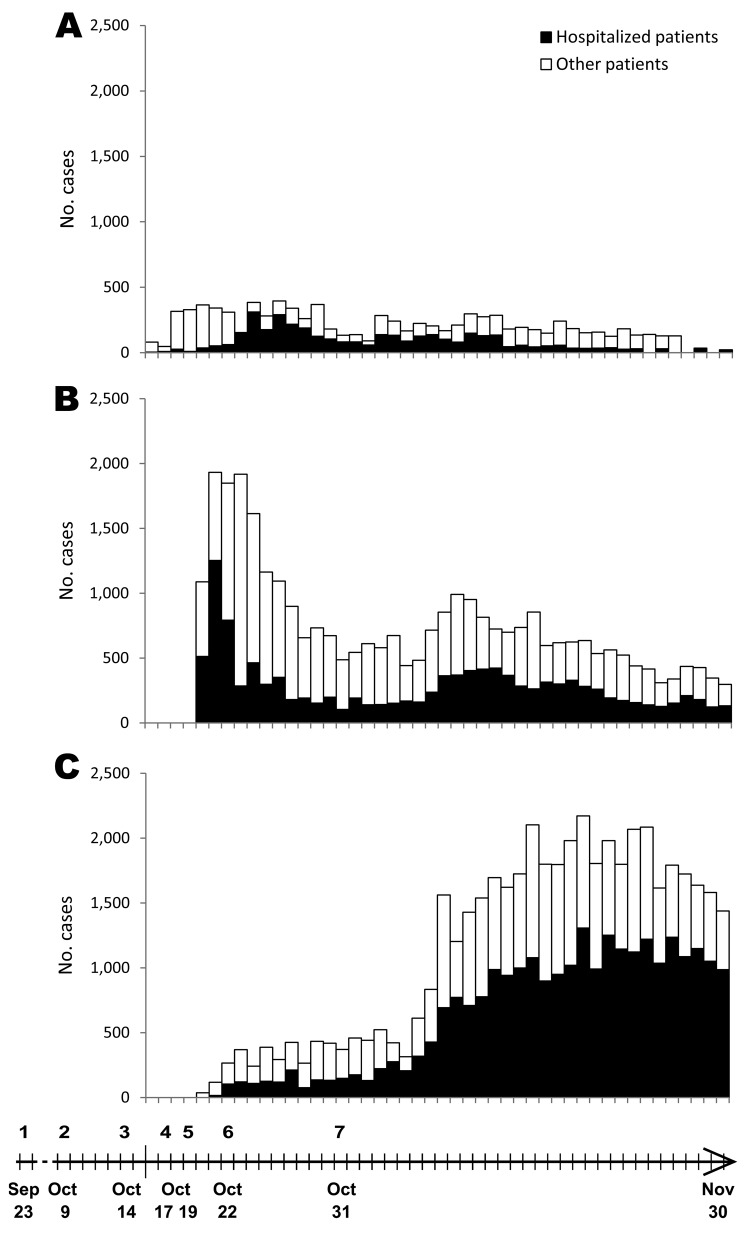
Cholera cases by date of onset of the epidemics and major related events, Haiti. A) Cases in Mirebalais, commune hosting the first cases of cholera; B) cases in seven communes simultaneously struck on October 20 (St-Marc, Dessalines, Desdunes, Grande Saline, Lestere, Petite-Rivière-de-l’Artibonite, Verrettes); C) cases in other communes. Timeline at bottom indicates 1) cholera outbreak in Kathmandu, Nepal; 2) first arrival of newly incoming Nepalese soldiers in Meille; 3) first cases in Meille; 4) first death registered in Mirebalais hospital (patient from Meille); 5) initiation of epidemic investigations and spread into the Artibonite delta; 6) epidemiologic confirmation of cholera cases in Meille; 7) United Nations camp sanitary dysfunction no longer observed.

On October 31, it was observed that sanitary deficiencies in the camp had been corrected. At the same time, daily incidence of cholera tended to decrease. Afterwards, incidence rose again to reach a second peak on November 10 (Figure 2).

## Spatiotemporal Modeling

**Figure 3:**
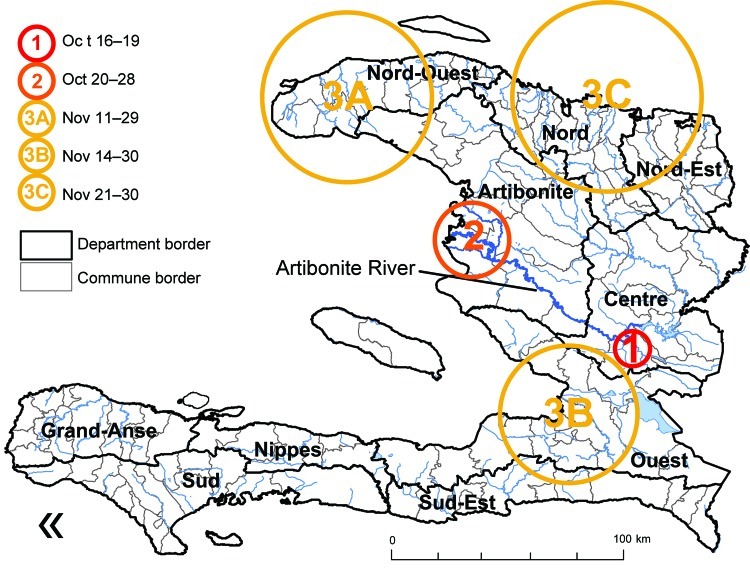
Spatiotemporal clusters of cholera cases, Haiti (results of SaTScan [Kulldorf, Cambridge, UK] analysis). The first cluster covered 1 commune, Mirebalais, October 16–19; the second cluster covered a few communes in or near the Artibonite delta during October 20–28; the next 3 clusters appeared in the North-West Department (A) during November 11–29, in the West Department (B) during November 14–30, and in the North and North-East Departments (C) during November 21–30. Other departments were affected later.

By using SaTScan (Kulldorf), several spatiotemporal clusters were identified (Figure 3): Mirebalais, October 16–19 (p<0.001), and in the Artibonite delta, October 20–28 (p<0.001). Overlapping staggered clusters occurred in the North-West (November 11–29; p<0.001); Port-au-Prince area (November 14–30; p<0.001); and North (November 21–30; p<0.001).

## Epidemic in Lower Artibonite

The start of the cholera epidemic was explosive in Lower Artibonite (communes of Grande Saline, St-Marc, Desdune, Petite-Rivière-de-l’Artibonite, Dessaline, and Verrettes). It peaked within 2 days and then decreased drastically until October 31 (Figure 2). On October 19, the departmental Direction of Health received a first alert from Bocozel (commune of St-Marc) where 3 children had died from acute watery diarrhea at school. The same day, clusters of patients with severe acute diarrhea and vomiting were admitted to a hospital in Dessalines, and deaths caused by severe diarrhea and vomiting were concomitantly reported in the community. During the next 24 hours, new alerts were registered from >10 health centers and hospitals located in each commune covering the lower course of the Artibonite River, from Desarmes (a locality 30 km from the sea) to the seashore (Figure 1). On October 21 at noon, <48 hours after the first alert, 3,020 cholera cases (including 1,766 hospitalizations) and 129 deaths were reported. No cholera cases had been reported in the Lower Artibonite area before October 19. In contrast, almost no cholera cases were recorded in the communes of Saut d’Eau (no case), Boucan Carre (no case), and La Chapelle (2 cases) on October 20 and 21. Only a few hamlets of these 3 communes located between Mirebalais and the Artibonite delta are crossed by the Artibonite River, so population density on its banks is low (Figure 1). Similarly, only 1 case, imported from Lower Artibonite, was reported in Gonaïve on October 20. Gonaïve is built in a floodplain adjacent to the Artibonite delta but watered by a different river running from the north.

The quasi-Poisson GAM model provided a fair goodness-of-fit with deviance explained of 89.4%. Adjusted for population and spatial location, location downstream of the Meille River and commune location in coastal plain were significant risk factors (risk ratios [RRs] 4.91 and 4.60, respectively) but the closeness to Mirebalais was not (Table 1).

**Table 1:** Adjusted risk ratio of cholera in each commune estimated by the generalized additive model, adjusted for population and spatial variability, Haiti, 2010*

Covariate	RR (95% CI)	p value
Location downstream of Meille River	4.91 (1.47–16.47)	0.012
Distance to Mirebalais, km	0.99 (0.94–1.04)	0.594
Presence of IDP camp	0.10 (0.01–1.12)	0.063
Commune located in coastal plain	4.60 (2.28–9.30)	0.0001

*RR, adjusted risk ratio; CI, confidence interval; IDP, internally displaced persons.

A strong correlation was found between the epidemic curves of the communes of the delta but not with that of Mirebalais (Table 2). The correlation was maximum (0.934) between St-Marc and Grande Saline, the 2 seashore communes bordering the main branch of the Artibonite River.

**Table 2:** Spearman rank correlation between the number of cases in the 8 communes of the Artibonite delta and corresponding p values, Haiti, October 16–31, 2010

Commune	Spearman correlation (p value)						
	L’Estère	Des Dunes	Verrettes	Grande Saline	Petite Rivière de l’Artibonite	Des Salines	St-Marc
Mirebalais	0.231(0.389)	0.527 (0.036)	0.276 (0.301)	0.480 (0.0.059)	0.563 (0.023)	0.361 (0.169)	0.459 (0.074)
St Marc	0.678 (0.004)	0.782 (<0.001)	0.652 (0.006)	0.934 (<0.001)	0.704 (0.002)	0.887 (<0.001)	
Des Salines	0.872 (<0.001)	0.713 (0.002)	0.465 (0.069)	0.818(<0.0001)	0.606 (0.013)		
Petite Rivière de l’Artibonite	0.672 (0.004)	0.675 (0.004)	0.586 (0.017)	0.648 (0.007)			
Grande Saline	0.600 (0.014)	0.848 (<0.001)	0.783 (<0.001)				
Verrettes	0.380 (0.147)	0.600 (0.014)					
Des Dunes	0.537 (0.032)						

## Spread Out of Artibonite Basin

On October 22, cholera cases were notified in 14 additional communes, most of them in the mountainous regions bordering the Artibonite plain and in Port-au-Prince. We visited several of these communes (Gonaïve, Ennery, Plaisance, Saint-Michel-de-l’Attalaye, and Port-au-Prince) and investigated the circumstances of the onset of cholera outbreaks. In each case, cholera started after the arrival of patients who fled from the ravaging epidemic in the Artibonite delta. There, numerous persons from bordering communes worked in rice fields, salt marshes, or road construction. The deadly epidemic provoked a panic that made them flee back home. Soon after, their communes of origin were experiencing outbreaks. In contrast, the southern half of Haiti remained relatively free of cholera after 6 weeks of epidemics (Figure 3). Spatiotemporal analysis identified slightly staggered clusters occurring from November 11, in North-West, Port-au-Prince, and North Departments, which are roughly equidistant from Artibonite delta. In the North, the largest epidemics occurred in the main cities located in floodplains, especially Cap Haitien and Gonaïve, but numerous deaths were recorded in the mountainous areas between Artibonite plain and northern coast. On November 20, almost 1 month after the first cases had been notified in Saint-Michel-de-l’Attalaye (139,000 inhabitants), we observed several small ongoing cholera outbreaks, striking 1 hamlet after another, leading to 941 cases (including 366 hospitalizations). Forty-one patients died in the hospital, and 110 died in the community. After 1 month, the death rate reached 1.08% in Saint-Michel-de-l’Attalaye.

In Port-au-Prince, the epidemic had 2 phases. For 15 days after the first patients arrived from Artibonite, the epidemic remained moderate with 76 daily cases on average from October 22 through November 5, causing only 77 hospitalizations. Then, the epidemic exploded in Cite-Soleil, a slum located in a floodplain close to the sea. However, after 6 weeks of epidemic, IDP camps were still relatively free of cholera. Despite the earthquake-related damages and the presence of many IDP camps, cholera struck less severely in Port-au-Prince, as demonstrated by incidence rate (0.51% until November 30, compared with 2.67% in Artibonite, 1.86% in Centre, 1.4% in North-West, and 0.89% in North) and cholera-related mortality rate (0.8 deaths/10,000 persons in Port-au-Prince, compared with 5.6/10,000 in Artibonite, 2/10,000 in Centre, 3.2/10,000 in North, and 2.8/10,000 in North-West). Living in the Port-au-Prince metropolitan area was associated with lower incidence (RR 0.51, 95% confidence interval 0.50–0.52; p<10^–7^) and lower mortality rates (RR 0.32, 95% confidence interval 0.28–0.37; p<10^–7^) than overall Haiti, even when considering unaffected departments.

## Discussion

Determining the origin and the means of spread of the cholera epidemic in Haiti was necessary to direct the cholera response, including lasting control of an indigenous bacterium and the fight for elimination of an accidentally imported disease, even if we acknowledge that the latter might secondarily become endemic. Putting an end to the controversy over the cholera origin could ease prevention and treatment by decreasing the distrust associated with the widespread suspicions of a cover-up of a deliberate importation of cholera (*15**,**16*). Demonstrating an imported origin would additionally compel international organizations to reappraise their procedures. Furthermore, it could help to contain disproportionate fear toward rice culture in the future, a phenomenon responsible for important crop losses this year (*17*). Notably, recent publications supporting an imported origin (*7*) did not worsen social unrest, contrary to what some dreaded (*18**–**20*).

Our epidemiologic study provides several additional arguments confirming an importation of cholera in Haiti. There was an exact correlation in time and places between the arrival of a Nepalese battalion from an area experiencing a cholera outbreak and the appearance of the first cases in Meille a few days after. The remoteness of Meille in central Haiti and the absence of report of other incomers make it unlikely that a cholera strain might have been brought there another way. DNA fingerprinting of *V. cholerae* isolates in Haiti (*1*) and genotyping (*7**,**21*) corroborate our findings because the fingerprinting and genotyping suggest an introduction from a distant source in a single event (*22*).

At the beginning, importation of the strain might have involved asymptomatic carriage by departing soldiers whose stools were not tested for the presence of *V. cholerae*, as the Nepalese army’s chief medical officer told the British Broadcasting Corporation (*23*). The risk for transmission associated with asymptomatic carriage has been known for decades (*24*), but asymptomatic patients typically shed bacteria in their stool at ≈10^3^ *V. cholerae* bacteria per gram of stool (*25*) and, by definition, have no diarrhea. This small level of shedding would be unlikely to cause interhuman contamination of persons outside the military camp having few contacts, if any, with MINUSTAH peacekeepers. By contrast, considering the presence of pipes pouring sewage from the MINUSTAH camp to the stream, the rapid dissemination of the disease in Meille and downstream, and the probable contamination of prisoners by the stream water, we believe that Meille River acted as the vector of cholera during the first days of the epidemic by carrying sufficient concentrations of the bacterium to induce cholera in persons who drank it. To our knowledge, only infectious doses >10^4^ bacteria were shown to produce mild patent infection in healthy volunteers, and higher doses are required to provoke severe infections (*26**,**27*). Reaching such doses in the Meille River is hardly compatible with the amount of bacteria excreted by asymptomatic carriers, whereas if 1 or several arriving soldiers were incubating the disease, they would have subsequently excreted diarrheal stools containing 10^10^–10^12^ bacteria per liter (*25*). We therefore believe that symptomatic cases occurred inside the MINUSTAH camp. The negativity of the repeated water samples disfavors the hypothesis of an environmental growth of the bacterium in the Meille stream even if the lack of use of molecular approaches precludes detection of low-level bacterial contamination. Alternatively, a contamination related to sewage discharge could have resulted in transient presence of the bacterium in the water, which could be easily missed by punctual samplings.

Our field investigations, as well as statistical analyses, showed that the contamination occurred simultaneously in the 7 communes of the lower course of the Artibonite River, an area covering 1,500 km^2^, >25 km from Meille. The abrupt upward epidemic curve in the communes bordering Artibonite dramatically contrasts with the progressive epidemic curve in the other communes of Haiti (Figure 2, panel B). In the latter, it took 19 days before the daily number of cases exceeded 1,000 (Figure 2, panel C). Suspected cholera was diagnosed in 7,232 patients during these 19 days. If the transmission in the communes bordering Artibonite had been similar to that of other communes, a comparable number of cases would have occurred in the days preceding the alert on October 20. So many cholera cases would not have remained unnoticed, all the more so as several health facilities of these communes were participating in the MSPP epidemiologic watch. The regression model indicates that the spread of cholera during the peak that occurred from October 20–28 was strongly linked to the Artibonite River and not to the proximity to Mirebalais, as would be expected for road-dependent propagation. This result, as well as the simultaneity of the outbreak onset in 7 communes of Lower Artibonite on October 19, is in accordance with contamination of the Artibonite River in a way that could infect thousands, and kill hundreds, of persons within a few days.

This hypothesis is also sustained by another early investigation during October 21–23 that showed that most affected persons worked or resided in rice fields alongside a stretch of the Artibonite River and that 67% drank untreated water from the river or canals (*1*). Cholera incubation varies from a few hours to 6 days (*26*), and the epidemic curve strongly suggests a rapid decrease of the contamination level in the river because the number of new cases and deaths dropped dramatically after only 2 days. A lasting phenomenon would have induced a continuing increase of incidence and a later peak. However, even for a few hours, contamination of a river such as the Artibonite requires a large amount of bacteria. For instance, to reach concentrations of 10^5^ *V. cholerae* bacteria per liter during only 3 hours in the Artibonite River, which usually flows >100 m^3^/s in October (*28*), >10^14^ bacteria are required. This level corresponds to the amount of bacteria in 1 m^3^ of rice-water stools harboring 10^11^ *V. cholerae* bacteria per liter. Notably, the fact that the peak in Mirebalais occurred later, on October 26, when daily incidence was dropping dramatically in Lower Artibonite also indicates that a specific mechanism was responsible for the onset of cholera in Lower Artibonite distinct from continuous spread from the primary focus.

Besides the particular circumstance that provoked the Artibonite’s outbreak, other factors may have played a role in the severity of the epidemic in Haiti: the absence of immunity among the population, the higher infectivity of epidemic strains shed in human rice-water stools than of environmental strains, and the role of hypervirulent variant strains in provoking epidemics (*24**,**29**,**30*). The recent sequencing of isolates from Haiti exhibited several structural variations that are hallmarks of the particularly virulent variant strains that have emerged in southern Asia (*7*).

Whatever its cause, this violent outbreak in Lower Artibonite provoked the flight of persons and resulted in a wave of epidemics that spread centrifugally and overwhelmed the nascent sanitarian response. This wave explains the difference between the delayed and progressive starting of epidemics in the south and the immediate impact of cholera in the north. Furthermore, after 6 weeks of epidemics, the IDP camps were still relatively free of cholera. Because the January earthquake led to population displacement, formation of camps, and overcrowding, numerous field actors considered that it was a favorable circumstance for a cholera epidemic. However, in most IDP camps, access to food, safe water, and sanitation was better than in neighboring wards (*2**,**31*). This low risk for epidemics after geophysical disaster was already reported in a study summarizing the epidemiologic consequences of >600 disasters (*32*).

Overall, this report highlights the importance of an accurate field investigation, especially when an epidemic strikes a previously unscathed area or evolves with unusual speed, to ensure an adequate targeting of the response by providing a feedback to the main field actors. Obviously, we have to be cautious with the interpretation that could be made from our results. Although they are compatible with the reports of several journalists who linked the epidemic with the dumping of a septic tank (*3*), the exact event that provoked the massive contamination of Lower Artibonite cannot be definitively deduced from an epidemiologic study. Rather, identifying the source and the responsibilities falls within the scope and competence of legal authorities. Nonetheless, this epidemic reminds us how critical the management of water and sewage is to prevent cholera spread. To avoid actual contamination or suspicion happening again, it will be important to rigorously ensure that the sewage of military camps is handled properly. Above all else, aid organizations should indeed avoid adding epidemic risk factors to those already existing and respect the fundamental principle of all assistance, which is initially not to harm―*primum non nocere*.
